# Carbon Dots for Corrosion Protection: A Systematic Review of Applications and Mechanisms

**DOI:** 10.3390/nano16080488

**Published:** 2026-04-20

**Authors:** Xiaochuan Liu, Jinlin Li, Shengbin Li, Chuang He, Haijie He

**Affiliations:** 1 School of Architectural Engineering, Yan’an University, Yan’an 716000, China; xiaochuanliu@yau.edu.cn (X.L.);; 2 School of Civil Engineering and Architecture, Taizhou University, Taizhou 318000, China; 3 College of Civil and Architectural Engineering, Zhejiang University, Hangzhou 310058, China

**Keywords:** carbon dots, corrosion protection, corrosion inhibitors, anticorrosive coatings, photoelectric cathodic protection

## Abstract

Carbon dots (CDs) have demonstrated promising application prospects in the field of corrosion protection due to their small size, excellent dispersibility, abundant and tunable surface functional groups, low cost, environmental friendliness, and unique fluorescence properties. However, existing reviews have predominantly focused on the synthesis and photoluminescence properties of CDs, lacking systematic integration and in-depth mechanistic analysis of their diverse applications in corrosion protection. This review systematically summarizes the recent research progress and underlying mechanisms of CDs in five key areas: corrosion inhibitors, anticorrosive coatings, photogenerated cathodic protection, chloride binding, and corrosion monitoring. As corrosion inhibitors, CDs form compact protective films on metal surfaces through synergistic physical and chemical adsorption. In anticorrosive coatings, CDs not only enhance the physical barrier effect but also impart intelligent functionalities such as self-healing and corrosion monitoring. In the field of photogenerated cathodic protection, CDs broaden the light absorption range of semiconductors and facilitate the separation of photogenerated carriers. As chloride binding promoters, CDs promote the formation of cement hydration products, thereby improving the durability of reinforced concrete structures. As sensing platforms, CDs enable early visual detection of corrosion through their specific fluorescence response to ions such as Fe^3+^. Despite significant progress, challenges remain in scalable preparation, practical application performance in complex environments, and multifunctional integration. This review systematically outlines the research advancements of CDs in corrosion protection, providing a practical reference for subsequent studies and engineering applications. Future research should focus on scalable synthesis, machine learning-assisted design, and the development of integrated multifunctional protection systems to promote the practical application of CDs in the field of corrosion protection.

## 1. Introduction

Corrosion, the functional degradation of materials (especially metals) due to chemical or electrochemical interactions with their environment, is a pervasive issue in industrial production, infrastructure, and marine engineering [1]. It not only causes substantial economic losses but can also trigger catastrophic safety accidents and environmental pollution [2,3,4]. Therefore, developing efficient, environmentally friendly, and durable corrosion protection technologies is of great significance for safeguarding national economies and public safety.

To combat corrosion, researchers have developed various protection strategies, primarily including corrosion inhibitors [5,6], protective coatings [7,8], cathodic protection [9,10], material modification (developing corrosion-resistant alloys such as weathering steel) [11,12], and surface treatment techniques (e.g., passivation) [13,14]. In recent years, with the advancement of nanotechnology, various nanomaterials have been introduced to enhance protective efficacy, such as graphene, two-dimensional transition metal dichalcogenides, MXenes, and boron nitride nanosheets [15,16].

**Figure 1:**
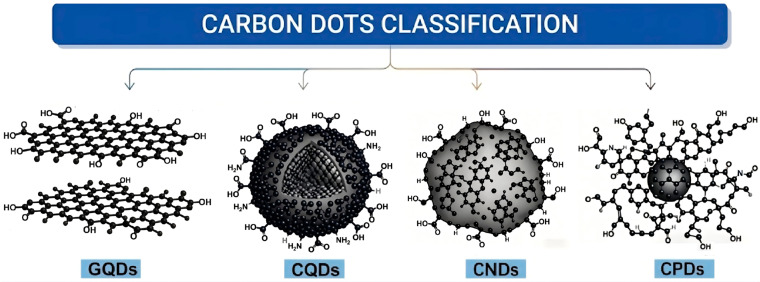
Classification of CDs.

Carbon dots (CDs) are an emerging class of zero-dimensional carbon-based nanomaterials, typically defined as quasi-spherical nanoparticles with a size less than 10 nm and primarily composed of carbon, which exhibit excellent fluorescence properties [17,18,19,20,21]. First discovered in 2004 during the purification of single-walled carbon nanotubes [22] and formally named in 2006 [23], CDs have since garnered widespread attention. Based on their core structure and formation mechanism, CDs are generally classified into four types: graphene quantum dots (GQDs), carbon quantum dots (CQDs), carbon nanodots (CNDs), and carbonized polymer dots (CPDs). Specifically, GQDs feature highly crystalline sp^2^ frameworks with typically fewer than three graphene layers; CQDs consist of multilayered, heteroatom-doped graphene domains with mixed sp^2^/sp^3^ hybridization; CNDs possess amorphous carbon cores with heavily oxidized surfaces; and CPDs are composed of carbon cores embedded in polymeric shells [24] (Figure 1). Leveraging their unique advantages, including excellent dispersibility, low cost, tunable surface functional groups, and environmental friendliness, CDs have shown great promise in the field of corrosion protection [1,25]. Currently, their applications have expanded into multiple sub-fields: as highly efficient corrosion inhibitors (effective in acidic, alkaline, and neutral solutions for various metals such as carbon steel, copper, and aluminum) [5,6,26]; as functional fillers in anticorrosive coatings to enhance barrier properties and self-healing capabilities [1,7]; as photoanode materials for photogenerated cathodic protection [27,28]; and even as chloride binding promoters to improve the resistance of reinforced concrete structures against chloride ion penetration in marine environments [29,30].

Although several reviews on CDs have been published, most focus on their synthesis and photoluminescence properties [13,24], or only discuss specific sub-domains of corrosion protection in isolation (e.g., only coatings or only inhibitors) [25,26]. More importantly, the field of CDs in corrosion protection is evolving rapidly, with a surge in publications in recent years. There is an urgent need for a comprehensive review that meticulously analyzes the specific application status in various sub-fields (corrosion inhibitors, anticorrosive coatings, photoanodes, etc.) and the underlying corrosion protection mechanisms, integrating dispersed research findings, distilling core advances, and providing clear direction for subsequent research.

Based on this, this paper aims to provide a systematic review of the application of CDs in corrosion protection, and is structured as follows: Section 2 provides an in-depth review of the application progress and inhibition mechanisms of CDs in the field of corrosion inhibitors; Section 3 systematically elaborates on the application of CDs in anticorrosive coatings and their related protection mechanisms; Section 4 discusses the application and mechanisms of CDs as photoanodes in photogenerated cathodic protection; Section 5 reviews the applications and mechanisms of CDs in other corrosion protection sub-fields (such as chloride binding promoters and corrosion sensing platforms); finally, Section 6 presents the conclusions and an outlook, identifying current research gaps in each sub-field and discussing future research priorities.

## 2. Progress on the Application and Mechanism of CDs in the Field of Corrosion Inhibitors

Corrosion inhibitors are chemical substances that, when present at an appropriate concentration in a corrosive medium, can significantly slow down or prevent metal corrosion. Due to their economy, efficiency, and ease of operation, they have become one of the most important means of metal protection. Traditional inhibitors such as chromates and nitrites, although efficient, face increasing application restrictions due to their high toxicity and environmental hazards. Therefore, the development of environmentally friendly and efficient novel green inhibitors has become a research hotspot. CDs, as an emerging class of zero-dimensional carbon-based nanomaterials, possess excellent water solubility, good chemical stability, low biotoxicity, low preparation cost, and abundant tunable surface functional groups. They have been proven to form effective adsorption films on metal surfaces, demonstrating great potential as green corrosion inhibitors. This chapter systematically reviews the application progress of CDs as inhibitors for various metals in different corrosive media and delves into their mechanisms of action.

### 2.1. Applications in a Variety of Corrosive Media

#### 2.1.1. Applications in Acidic Media

The inhibition effect of CDs on carbon steel in acidic media was first confirmed in 2015. Zhou and colleagues [31] used an electrochemical oxidation method with a graphite rod as the electrode and an aqueous solution of triammonium citrate as the electrolyte to prepare water-soluble GQDs with diameters of 5–30 nm and oxygen-containing functional groups (–OH, –COOH). They found that at an optimal concentration of 200 mg/L, these GQDs exhibited a good inhibition effect (78.6%) on carbon steel in 1 mol/L hydrochloric acid, as determined by electrochemical impedance spectroscopy (EIS), acting as a mixed-type inhibitor. This laid the foundation for the application of CDs in the field of corrosion inhibitors. In general, the morphology (e.g., particle size and height) of CDs is typically characterized by transmission electron microscopy (TEM) and atomic force microscopy (AFM), while their surface functional groups are identified by Fourier transform infrared spectroscopy (FTIR) and X-ray photoelectron spectroscopy (XPS). The inhibition performance of CDs is commonly evaluated through weight loss measurements, EIS, and potentiodynamic polarization (PDP) curves.

**Figure 2:**
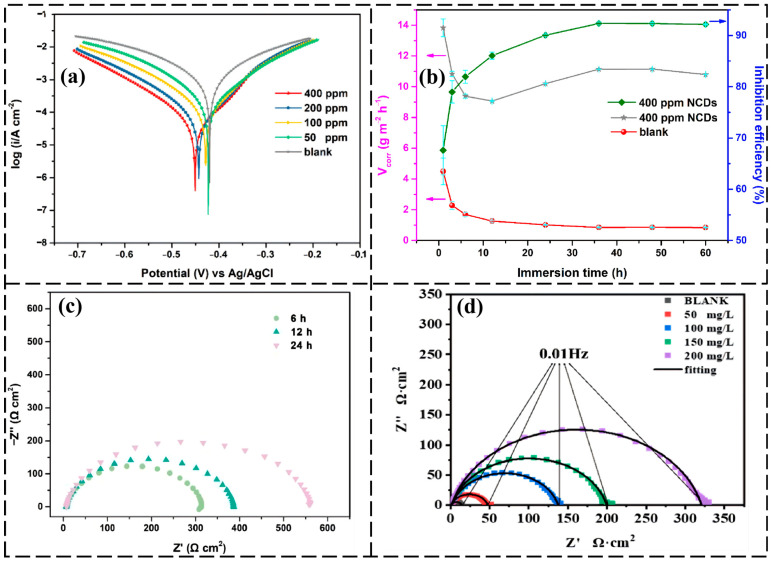
Inhibition performance and corrosion kinetics of NCDs for Q235 carbon steel in 1 M HCl medium: (**a**) PDP curves of carbon steel in blank solution and with different concentrations of NCDs (50, 100, 200, 400 ppm). Reprinted from Ref. [35]; (**b**) Variation of corrosion rate (left axis) and inhibition efficiency (right axis) of carbon steel with immersion time in blank solution and with 400 ppm NCDs. Reprinted from Ref. [35]; (**c**) Nyquist plots of electrochemical impedance spectroscopy for Q235 carbon steel after various durations of exposure to 1 M HCl solution in the presence of 400 ppm NCDs. Reprinted from Ref. [35]; (**d**) Nyquist plots of electrochemical impedance spectroscopy for carbon steel in blank solution and with different concentrations of NCDs. Reprinted from Ref. [42].

Subsequently, researchers began exploring heteroatom doping strategies to further enhance the inhibition performance of CDs [32,33]. Inspired by the excellent water solubility and low cytotoxicity of NCDs (with an average size of about 3 nm and surface groups including –OH, –COOH, –NH–, and C–N–C) prepared from the antibiotic aminosalicylic acid by a facile solvothermal method, Zhao’s group [32] applied them as environmentally friendly inhibitors for the corrosion protection of Q235 carbon steel in 1 M HCl solution. Through PDP plots and weight loss measurements, they found that the inhibition efficiency increased with increasing CD concentration and prolonged immersion time, reaching a maximum value of 93–96% when the adsorption of CDs on the steel surface reached equilibrium at an optimal concentration. This is attributed to the formation of an adsorption film on the steel surface. The adsorption behavior followed the Langmuir isotherm, and the efficiency increased with concentration and immersion time. Gui and colleagues [34] further extended the research to sulfuric acid media, synthesizing nitrogen-doped functionalized CDs (NCDs) via a one-step hydrothermal method using citric acid and urea as precursors. They found that in 0.5 M H_2_SO_4_, only 30 mg/L of NCDs achieved an inhibition efficiency exceeding 95% for carbon steel at 298 K, also conforming to the Langmuir adsorption model. Inspired by these studies, Zheng and colleagues [35] prepared NCDs using the green material dopamine as a precursor by a hydrothermal method and systematically investigated their inhibition behavior on Q235 carbon steel in 1 M HCl. Electrochemical tests indicated that NCDs, as a mixed-type inhibitor, effectively retarded acid corrosion, achieving a high efficiency of 96.1% at 400 ppm at room temperature (Figure 2a). Weight loss experiments showed that in the presence of 400 ppm NCDs, the inhibition efficiency increased with prolonged immersion time and elevated temperature (298–328 K) (Figure 2b,c), attributed to the strong adsorption of NCDs on the steel surface. These results fully highlighted the enhancing effect of nitrogen doping on the inhibition performance of CDs.

To further verify this enhancement effect at the mechanistic level, He and colleagues [36] comparatively studied the inhibition performance of undoped and nitrogen-doped GQDs (GQDs and NGQDs) on carbon steel in the 1 M HCl corrosive medium. The study found that at a concentration of 200 mg/L, the inhibition efficiency of GQDs was 83.32%, while that of NGQDs reached 89.25%. Density functional theory and molecular dynamics simulations indicated that N doping endowed N-GQDs with better electron-donating ability, resulting in stronger adsorption on the steel substrate compared to GQDs. Notably, even with N doping, the performance of CD inhibitors can vary significantly depending on the type of doping atom. Xu’s group [37] synthesized NCDs containing different nitrogen forms (amino-N, pyrrolic-N, graphitic-N) using a two-step hydrothermal method and found that NCDs with the highest pyrrolic-N content (4.10 at.%) achieved an inhibition efficiency of 96.63% at 200 mg/L, Besides N doping, S doping also showed excellent performance. Cui’s team [38] prepared NCDs and nitrogen–sulfur co-doped carbon dots (N,SCDs) via a one-step hydrothermal method and compared the inhibition efficiencies of single-element and dual-element doping. After 1 h of immersion at 200 ppm, the inhibition efficiency of NCDs was approximately 87.9%, while that of N,SCDs reached 96.4%. Molecular dynamics simulations suggested that the multi-anchoring adsorption of N,SCDs on the carbon steel surface led to stronger interactions with the Fe substrate, thus resulting in superior inhibition performance. Further research revealed that the doping ratio also affects performance. Li and colleagues [39] synthesized N,SCDs using different ratios of ammonium citrate and methionine. When the ratio was 90:10, the highest inhibition efficiency for carbon steel at 200 mg/L was achieved, reducing the corrosion current density by 1–2 orders of magnitude, with adsorption involving physicochemical interactions.

Although the use of small molecule precursors to prepare heteroatom-doped CDs yields significant effects, their potential environmental risks cannot be ignored. Therefore, researchers began utilizing environmentally friendly and readily available biomass as precursors. He’s team [40] first prepared biomass-derived CDs (LCDs) from litchi leaves via a green hydrothermal treatment. The as-prepared LCDs exhibited a size distribution of 2.56–6.45 nm (average ~4.24 nm) and contained surface functional groups including O–H/N–H, C=O, C–N, and C–O. At only 200 mg/L, they achieved an inhibition efficiency of 98.06% for Q235 carbon steel in 1 M HCl. Subsequently, biomass-derived CQDs (BCQDs) prepared by Feng’s group [41] showed an efficiency of 94.1% at 308 K and 200 mg/L, with the mechanism involving self-aggregation and adsorption forming a dense protective film. Ma’s team [42] prepared eco-friendly self-doped CQDs (ZCQDs) from Zanthoxylum bungeanum leaves via solid-phase pyrolysis, achieving a high yield of 25%. At 200 mg/L, the efficiency reached 95.98% (Figure 2d), providing effective protection for at least 132 h. Guo’s team [43] synthesized NCDs using biomass waste coffee grounds as the carbon source and polyaspartic acid as the nitrogen source. At an extremely low concentration of 10 mg/L, the efficiency reached 93.9%, forming a stable protective film through chemical coordination with Fe atoms. These studies indicate that biomass, being widely available and environmentally friendly, is an ideal precursor for preparing high-performance CD inhibitors.

Besides carbon steel, CDs also show protective potential for other metals in acidic media. Wang’s team [44] prepared NCDs via a hydrothermal method that effectively inhibited copper corrosion in H_2_SO_4_ medium. The NCDs formed an adsorption film on the copper substrate, acting as a barrier layer and preventing the transport of corrosive particles. However, research on the inhibition effect of CDs on other non-ferrous metals (such as aluminum, zinc) in acidic media is still scarce and requires further exploration.

#### 2.1.2. Applications in Neutral/Saline Media

In neutral environments containing chloride ions (such as groundwater, seawater), chloride ions can easily induce pitting corrosion on metals, seriously threatening the long-term service safety of metal facilities. Research on the application of CDs in such media has gradually increased in recent years, providing new ideas for solving practical engineering corrosion problems. In 2019, Zhao’s group [45] first applied imidazole ionic liquid and citric acid-based CDs for the corrosion protection of Q235 steel in 3.5 wt.% NaCl solution. They found that this CD inhibitor was also effective in NaCl solution, with the mechanism conforming to the Langmuir adsorption model, involving physicochemical adsorption. Building on this, Ye’s group [46] further optimized cost and efficiency. NCDs prepared via a hydrothermal method achieved an inhibition efficiency of 88.96% for Q235 steel in 3.5 wt.% NaCl at a concentration of 200 mg/L. The NCDs formed an adsorption film on the steel surface, effectively resisting Cl^−^ attack.

Beyond carbon steel, significant progress has also been made in the inhibition of other metals by CDs in neutral media. Zhang et al. [47] prepared highly nitrogen-doped CDs (N/C atomic ratio up to 23.95%) using melamine and fructose as precursors via a solvothermal method, which exhibited a spherical morphology with an average size of about 7 nm and surface groups such as N–H, C–H, C=O, and C–N. These achieved an inhibition efficiency as high as 96.1% for copper in 3 wt.% NaCl solution (200 mg/L). The mechanism involved the adsorption of highly N-doped CDs on the copper surface and chelation with copper ions to form a dense protective film. Wang’s group [48] used waste polypropylene medical masks as a single precursor to prepare CDs by a one-pot in situ acid oxidation hydrothermal strategy, achieving dual functions of ratiometric fluorescence sensing and corrosion inhibition at nearly 100% yield. At a concentration of 200 mg/L, the inhibition efficiency for copper substrate in 3.5 wt.% NaCl reached 94.01%, outperforming most reports. In addition, the CDs can be used for the fluorescence detection of Cr(VI) with a detection limit of 24 nM. These results demonstrate the dual functionality of the CDs; i.e., corrosion protection and heavy metal ion sensing, rather than integrated monitoring during inhibition. In addition to copper, CDs have also been applied as corrosion inhibitors for magnesium alloys in NaCl solution. Zhang et al. [49] synthesized N/S co-doped CDs from sesame straw via a pyrolysis method. The CDs had a graphite-like structure of several nanometers in size, with surface groups including O–H, N–H, C–N, C–O–C, and aromatic C–H (Figure 2). For AZ31 Mg alloy in 3.5 wt.% NaCl, they achieved an inhibition efficiency of 83.8% at 200 mg/L (determined by EIS). These studies indicate that the application of CDs in neutral chloride-containing environments has gradually expanded from initial feasibility verification to performance optimization and functional integration through heteroatom doping and structural design, showing promising prospects. Currently, the protective performance of CD inhibitors for light metals such as aluminum in neutral saline solutions remains to be explored and represents a worthwhile future research direction.

#### 2.1.3. Applications in Alkaline Media

In alkaline environments, the problem of chloride-induced corrosion in concrete structures is particularly prominent. Chloride ions penetrating the concrete can destroy the passive film on reinforcing steel, induce pitting corrosion, and subsequently lead to steel expansion and concrete cracking, severely threatening the durability and safety of the structure. Against this background, He’s team [50] first introduced CDs as corrosion inhibitors into chloride-contaminated alkaline systems, systematically studying their protective performance on carbon steel in simulated concrete pore solution (SCPS). The results showed that adding only 400 mg/L of CDs achieved a long-term inhibition efficiency as high as 99.2%, demonstrating excellent protective effects. This research not only provided a novel and efficient inhibitor for corrosion control of reinforced concrete structures in chloride environments, but also expanded a new direction for the application of CDs in alkaline media. To date, research on the inhibition of other metals such as aluminum and copper by CDs in alkaline environments has not been reported and represents an important area for future exploration.

Furthermore, as research deepens, traditional trial-and-error methods are increasingly unable to meet the demands of developing efficient inhibitors, being both resource-intensive and potentially environmentally burdensome. In recent years, data-driven machine learning methods have provided a new paradigm for the controlled synthesis and performance prediction of CD inhibitors. In 2024, He’s team [51] first introduced machine learning into the research field of CD inhibitors, aiming to accurately predict the inhibition efficiency of CDs and optimize their synthesis routes. The research team collected 102 sets of CD synthesis parameters and inhibition efficiency data from the published literature and self-built experiments, constructing a dedicated dataset. After training and evaluating various machine learning models, the random forest regression model was ultimately selected, exhibiting the lowest root mean square error and mean absolute error, and the highest coefficient of determination. The study showed that the model could comprehensively reveal the structure–activity relationship between hydrothermal synthesis parameters and inhibition efficiency. With the assistance of a genetic algorithm, it not only achieved accurate prediction of inhibition efficiency (error < 10%) but also successfully optimized CD synthesis conditions. This work first verified the feasibility of machine learning technology in guiding the optimization of synthesis conditions for CD inhibitors, potentially significantly shortening the R&D cycle and reducing costs, providing new ideas for the sustainable development and clean production of green inhibitors. In the future, by further enriching databases and integrating multi-scale theoretical simulations, machine learning is expected to become a key tool for the design of high-performance CD inhibitors.

### 2.2. Inhibition Mechanism of CDs Inhibitors

Based on existing research, the excellent inhibition performance of CDs primarily stems from the formation of a protective adsorption film on the metal surface. Their mechanism of action can be summarized in the following aspects.

Firstly, CDs form a physical barrier through adsorption on the metal surface. Due to their nanoscale size and good dispersibility, CDs can uniformly adsorb onto the metal surface, forming a dense protective film that effectively blocks the contact of corrosive media (H^+^, Cl^–^, H_2_O, O_2_) with the substrate [31,34,36,45]. This adsorption behavior typically follows the Langmuir isotherm adsorption model [34,35,43,45,46,51], indicating that CDs form a monolayer coverage on the metal surface, with coverage increasing with concentration [42].

Secondly, the adsorption driving force originates from the synergistic effect of physical adsorption and chemical adsorption. The abundant oxygen-containing (–OH, –COOH) and nitrogen-containing (–NH_2_) functional groups on the CD surface are key to adsorption. On one hand, these polar groups can undergo physical adsorption with charged metal surfaces through electrostatic interactions [42,43,47]. On the other hand, heteroatoms (N, S, O) in CDs possess lone pair electrons, and their aromatic ring structures have delocalized π-electrons, which can form coordination bonds with the empty d-orbitals of metals like Fe and Cu, resulting in chemical adsorption. Chemical adsorption is stronger and forms the basis for a stable, durable protective film.

**Figure 3:**
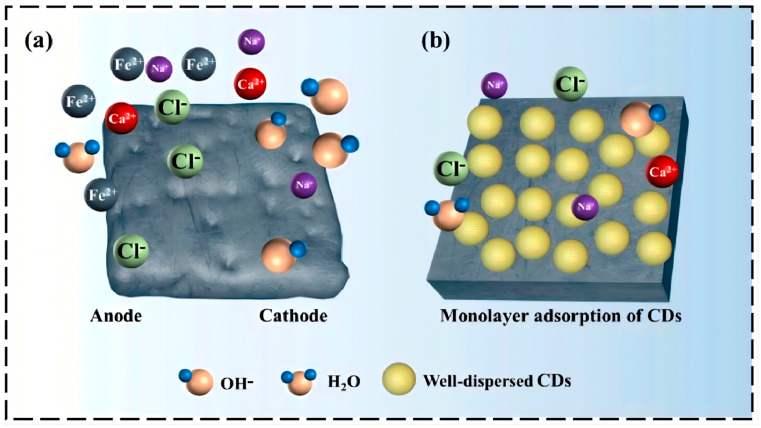
Corrosion inhibition mechanism of carbon steel in chloride-contaminated SCPS: (**a**) Schematic diagram of carbon steel samples in chloride-contaminated SCPS without CDs. Reprinted with permission form Ref. [50]. Copyright 2024, Elsevier; (**b**) Schematic diagram of carbon steel samples in chloride-contaminated SCPS with CDs. Reprinted with permission form Ref. [50]. Copyright 2024, Elsevier.

Thirdly, heteroatom doping can significantly enhance the adsorption capacity of CDs. Doping atoms (such as N, S) can alter the electron cloud distribution and energy level structure of CDs. N doping enhances the electron-donating ability of CDs, making them more prone to form coordination bonds with metals. The influence of different doping types varies; pyrrolic-N, due to its lone pair electrons participating in conjugation, can more effectively enhance the reactivity of CDs and their parallel adsorption on the metal surface (Figure 3a,b) [37]. Multi-element (N, S) co-doping provides multiple adsorption sites, enabling multi-anchoring and forming a more stable protective layer [38,39]. Optimization of the doping ratio further improves the inhibition performance of CDs [39].

Fourthly, CDs can promote the formation of a passive film or participate in constructing a composite protective layer themselves. In alkaline chloride environments, the adsorption of CDs can stabilize the original passive film on the metal surface (such as γ-FeOOH) or provide favorable conditions for repassivation [50]. In neutral saline solutions, highly nitrogen-doped CDs can chelate with copper ions to form a dense protective layer [47,48]. Furthermore, at high concentrations, CDs not only form a monolayer adsorption on the metal surface but also form thicker, denser protective layers through layered adsorption, further enhancing the barrier effect [42].

In summary, as a novel type of green corrosion inhibitor, CDs have demonstrated excellent protective performance for various metals such as carbon steel and copper in acidic, alkaline, and neutral media. Their mechanism of action primarily involves forming a dense protective barrier through physical/chemical adsorption on the metal surface. Heteroatom doping and the selection of biomass precursors are key strategies for optimizing their performance and achieving sustainable development. Although significant progress has been made, further in-depth exploration is needed regarding their inhibition behavior in complex multiphase media, compatibility with engineering materials like concrete, machine learning-assisted intelligent design, and large-scale low-cost preparation processes to promote their widespread application in practical industrial environments.

## 3. Progress on the Application and Protective Mechanisms of CDs in Anticorrosive Coatings

Anticorrosive coatings are one of the most direct and widely used methods for preventing metal corrosion, forming a physical barrier on the metal surface that isolates water, oxygen, and aggressive ions. However, traditional organic coatings are prone to defects such as micropores and cracks during long-term service, leading to a decline in protective performance. CDs, owing to their unique advantages, including small size (<10 nm), excellent dispersibility, abundant and tunable surface functional groups, good fluorescence properties, and low cost, have been widely introduced into coating systems. CDs can not only enhance the density and adhesion of coatings but also endow them with intelligent functions such as self-healing and corrosion monitoring, making them a research hotspot in the field of anticorrosive coatings. This chapter systematically reviews the application progress of CDs in various types of anticorrosive coatings and delves into their mechanisms for enhancing corrosion protection.

### 3.1. Applications of CDs in Anticorrosive Coatings

Research on the application of CDs in anticorrosive coatings began in 2017. Kang’s team [52] first introduced CDs into polymer coatings, discovering that they not only significantly enhanced anticorrosive performance but also imparted self-healing capabilities to the coatings. In detail, they blended CDs with an average particle size of about 5 nm and surface functional groups including –OH, –COOH, –NH_2_, and –CONH_2_ (as determined by titration and XPS) into common polymers such as polymethacrylate and polyurethane, finding that the interfacial bonding (covalent bonds, hydrogen bonds, and van der Waals forces) between the CDs and the polymer matrix was the driving force behind the self-healing process. By comparing the tensile strength of pristine composites with those that were fractured and then healed, and by observing the self-healing behavior of intentionally introduced scratches, they confirmed the self-healing property of the composite coatings, which also exhibited superior anticorrosive performance compared to pure polymer coatings.

Subsequently, researchers further improved the compatibility and anticorrosive effect of CDs in coatings through functionalization modification. Pourhashem et al. [53] functionalized GQDs (f-GQDs) using a silane coupling agent to enhance their structural compatibility with solvent-based epoxy. The resulting f-GQDs contain –OH, C=O, C=C, –NH_2_, C–N, and newly introduced Si–O–C and Si–O–Si groups. Electrochemical tests showed that the addition of f-GQDs effectively improved the corrosion protection performance of the epoxy coating on mild steel substrate, which is attributed to the good barrier effect formed within the coating. Zhao’s team [54] prepared NCDs with size of 4−6 nm via a solvothermal method and incorporated them into a waterborne epoxy coating on Q235 steel. Electrochemical impedance spectroscopy revealed that the impedance modulus of the epoxy coating containing 2.0% NCDs was 364 times higher than that of the blank epoxy after 800 h of immersion. Mechanistic analysis indicated that the abundant functional groups on the NCD surface formed connections with the waterborne epoxy, suppressing the enlargement of pores and reducing oxygen diffusion, thereby delaying substrate corrosion. Apart from their application in steel coating corrosion protection, CDs have also been employed for enhancing the corrosion resistance of magnesium alloy coatings. For example, Jiang et al. [55] fabricated an N-GQDs/PMTMS composite coating on AZ91D Mg alloy via electrodeposition and silane treatment. The N-GQDs with the size of 2–10 nm were synthesized via a hydrothermal method using citric acid and urea as raw materials and exhibited high crystallinity and blue fluorescence. The composite coating consisted of a ~7 μm thick N-GQDs inner layer and a ~12 μm thick PMTMS top layer. Compared with the bare Mg alloy, the composite coating showed a noticeable enhancement in corrosion resistance, characterized by a polarization resistance (*R*_s_) more than six times that of the bare alloy. The constant phase element decreased by orders of magnitude (bare Mg: 1.0 × 10^−5^, N-GQDs coating: 4.5 × 10^−6^, N-GQDs/PMTMS: 4.3 × 10^−9^ Ω^−1^·s^n^·cm^−2^). Similarly, CDs have been combined with polydopamine for Mg alloy protection. Zhang et al. [56] fabricated an N-CDs/PDA composite coating on AZ91D Mg alloy via electrodeposition and dip coating. The corrosion resistance of the N-CDs coating increased with larger particle size. The optimal N-CDs-8/PDA coating showed an extremely low corrosion current density (1.6 × 10^−7^ A·cm^−2^) and exhibited self-healing behavior due to the PDA layer, significantly outperforming the bare Mg alloy.

The self-healing function represents an important development direction for CDs-modified coatings. Zhao’s team [57] further composited functionalized CDs with graphene, preparing a CDs-modified graphene/epoxy coating (CDs-G/EP) via π-π interactions. Structural characterization showed that the introduction of CDs significantly enhanced the dispersion and interfacial compatibility of graphene in the epoxy. Electrochemical results indicated that the coating containing 0.5 wt.% CDs-G exhibited excellent protective performance, attributed to the physical barrier of highly dispersed graphene and the self-healing ability of the CDs. After 50 d of immersion, its oxygen permeability coefficient and water absorption were only 4.27 × 10^−13^ cm^3^ cm cm^−2^ s^−1^ Pa^−1^ and 4.4%, respectively. Qiang’s team [2] used CQDs derived from fruit juice to modify graphitic carbon nitride (g-C_3_N_4_). The resulting CQDs@g-C_3_N_4_ composite exhibited good compatibility in epoxy and significantly enhanced the barrier performance of the coating. Simultaneously, the adsorption of CQDs on the metal endowed the coating with active protection performance, and their fluorescence properties could be used to detect micro-cracks in the coating, achieving the integration of active/passive self-healing and early corrosion monitoring.

The synergistic modification of CDs with two-dimensional materials has become a recent hotspot. Inspired by the predation behavior of the Venus flytrap, Zhang’s team [58] co-modified Ti_3_C_2_T_X_ MXene with CDs and phenanthroline derivatives, preparing a PCD-MX/WEP coating with excellent dispersion and stress closure functions. After 90 d of immersion in 3.5 wt.% NaCl solution, the low-frequency impedance modulus of this coating still reached 5.83 × 10^8^ Ω·cm^2^, which was one to two orders of magnitude higher than that of the blank waterborne epoxy. After being buried in outdoor soil for 70 d, it remained uncorroded, with an impedance modulus of 3.61 × 10^7^ Ω·cm^2^. Density functional theory calculations indicated that both the [Fe(Phen)_3_]^2+^ and CD@Fe^3+^ complexes were thermodynamically stable, while hydrogen bonding between PCD-MX and the epoxy resin ensured good interfacial compatibility. The self-healing ability of the coating originated from the rapid formation of the [Fe(Phen)_3_]^2+^ complex within the PCD-MX, and the in situ capture of metal cations by the phenanthroline groups could significantly inhibit the generation of corrosion products. Zhao’s team [59] also introduced CDs into layered double hydroxides (LDH), preparing LDH-Cdot nanohybrids. Carboxyl-rich CDs inserted between the LDH layers significantly improved the dispersion of LDH in water. After incorporating LDH-Cdot into waterborne epoxy, the coating maintained an impedance modulus of 4.05 × 10^8^ Ω·cm^2^ after 90 d of immersion. Local electrochemical impedance and corrosion product analysis indicated that the CDs anchored on the LDH could coordinate with metal ions, promoting the formation of a passive layer and effectively slowing corrosion kinetics. Daksha et al. [60] were the first to synthesize CDs-functionalized graphene oxide (CDs-f-GO) via a one-step method and incorporated it into an epoxy coating. This hybrid material not only exhibited excitation-dependent fluorescence properties (distinct from non-fluorescent GO) but also significantly enhanced the anticorrosive performance of the coating. Salt spray tests showed minimal corrosion blistering on scratched coatings, indicating excellent resistance to electrolyte penetration. Simultaneously, the coating enabled real-time failure detection under UV light, with scratches clearly visible, offering the possibility for early defect detection in steel structures. Dai’s team [61] and Zhu’s team [62] used CDs to intercalate and modify α-zirconium phosphate (α-ZrP), preparing LCDs-ZrP and PCDs@ZrP hybrids, respectively, which were then incorporated into waterborne epoxy coatings. The introduction of CDs not only prolonged the diffusion path of corrosive media through the maze effect but also allowed their surface groups (–SH, –OH, –NH_2_, –COOH) to coordinate with the steel substrate, forming a protective film and synergistically enhancing the coating’s anticorrosive performance. The impedance value of the LCDs-ZrP/WE coating reached 9.97 × 10^9^ Ω·cm^2^ after 28 d of immersion, far exceeding that of the pure WE coating (4.74 × 10^6^ Ω·cm^2^). The |Z|_0.01_ Hz value of the PCDs@ZrP coating in 3.5 wt.% NaCl after 30 d of immersion was three orders of magnitude higher than that of the pure WEP coating.

**Figure 4:**
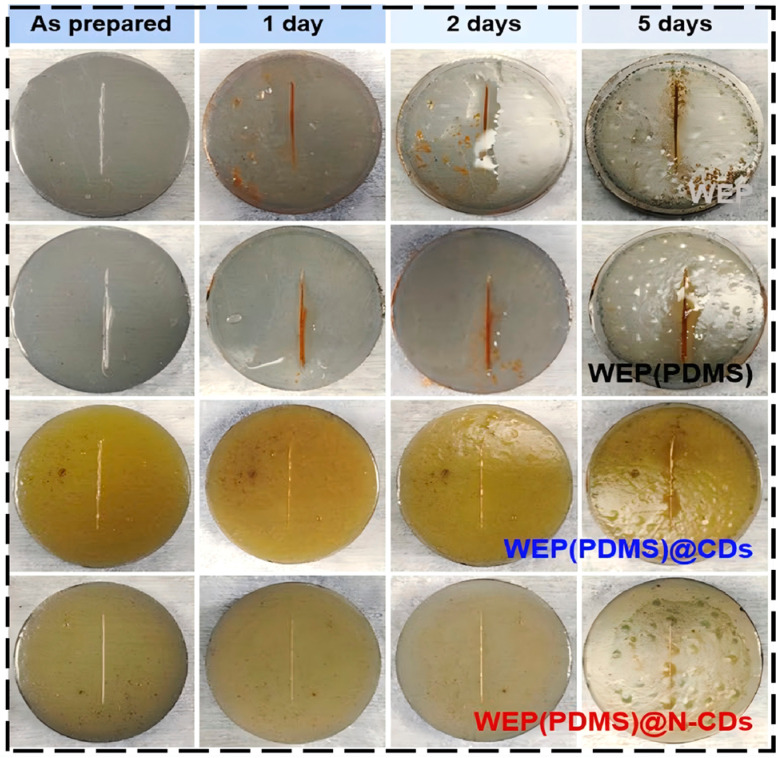
Self-Healing Properties of CD-Modified Coatings. Reprinted from Ref. [65].

CDs have also been used to endow coatings with functions such as lubrication and photothermal response. Wolk et al. [63] synthesized dodecylamine edge-functionalized few-layer graphene oxide quantum dots, which exhibited excellent solubility in various organic solvents. They could be sprayed onto steel surfaces to form a thin film, reducing the friction coefficient from 0.17 to 0.11 and showing a significant corrosion inhibition effect. Zhou’s team [64] anchored NCDs onto Ti_3_C_2_T_x_ MXene, preparing a CDs@Ti_3_C_2_T_x_/EP composite coating. After 70 d of immersion in 3.5 wt.% NaCl, the low-frequency impedance of this coating still reached 1.38 × 10^11^ Ω·cm^2^, while the friction coefficient was reduced to 0.234 and the wear rate was as low as 4.87 × 10^−4^ mm^3^/Nm, achieving the integration of long-term anticorrosion and lubrication. Jin et al. [65] synthesized NCDs with a polymer–carbon core hybrid structure. After incorporating them into a waterborne epoxy coating, the friction coefficient of the coating decreased from 0.760 to 0.049 (a reduction of 93.6%), while the low-frequency impedance modulus reached 3.5 × 10^7^ Ω·cm^2^. The performance improvement was attributed to the abundant polymer branches on the N-CD surface, which increased the crosslinking density of the coating, enabling dynamic repair during friction and enhancing the barrier effect (Figure 4). Wang’s team [66] designed a dual-chamber microcapsule (particle size about 5 μm) with furfurylamine-modified polystyrene–acrylic acid as the shell and the photosensitizer CDs as a Pickering emulsifier. After incorporation into epoxy resin, the composite material could heat up to 50 °C within 60 s, achieving photothermal-responsive self-healing. The epoxy coating containing 2 wt.% microcapsules exhibited excellent strength self-healing efficiency, modulus, toughness, and fatigue resistance, with EIS analysis confirming its long-term corrosion resistance.

CDs have also been extended to other coating systems. Li et al. [67] doped GQDs into polyphenylene sulfide, preparing a GQDs/PPS composite coating. The coating containing 5% GQDs exhibited the largest water contact angle, good hydrophobicity, and excellent thermal stability (weight loss rate of only 23.37% at 800 °C). Electrochemical tests showed that it exhibited the best corrosion resistance in H_2_SO_4_, NaOH, and NaCl solutions, with a corrosion current as low as 5.45 × 10^−10^ A/cm^2^. Duarte et al. [68] were the first to apply CQDs to metallic tin coatings, preparing Sn-CD composite coatings on mild steel via galvanostatic electrodeposition. The study found that at a CD concentration of 0.05 g/L in the bath, the coating grew more uniformly and exhibited enhanced oxidation resistance. XPS analysis showed that the pure tin coating oxidized more severely than the composite coating, confirming that the addition of CDs improved the material’s corrosion resistance. Xiong’s team [69] applied CDs for the protection of anodes in aqueous zinc-ion batteries, constructing a TiO_2_/NCDs hybrid protective layer. The –OH, –COOH, and –NH_2_ groups on the NCD surface provided zincophilic nucleation sites, resulting in a low zinc deposition overpotential of 28 mV and guiding zinc to deposit in a petal-like morphology. This protective layer effectively inhibited zinc dendrite growth and interfacial corrosion, and the Zn-TiO_2_/NCDs anode achieved a long cycle life of 1500 h at 5 mA cm^−2^ and 2.5 mAh cm^−2^.

Furthermore, CDs can endow coatings with various additional functions, such as lubrication [63,64,65], fluorescence monitoring [2,60], photothermal response [66], and dendrite inhibition [69]. These functions drive the development of coatings towards intelligence and multifunctionality.

### 3.2. Mechanisms of CDs in Enhancing Coating Anticorrosive Performance

Based on existing research, the enhancing effects of CDs on coating anticorrosive performance can be summarized in the following aspects.

**Figure 5:**
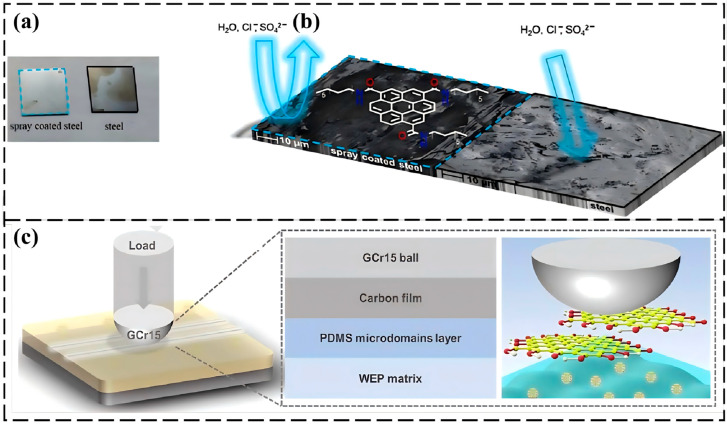
Multi-scale protection mechanisms of CDs-based anticorrosive systems: (**a**) Visual comparison of corrosion morphology between CDs-modified steel and bare steel, verifying the practical protective effect of CDs. Reprinted from Ref. [63]; (**b**) Interfacial inhibition mechanism of functionalized CDs forming a dense film on steel, blocking corrosive ion penetration. Reprinted from Ref. [63]; (**c**) Dual function of CDs in waterborne epoxy coatings, showing a physical barrier against corrosive media and interfacial bonding for coating self-healing. Reprinted from Ref. [65].

Firstly, CDs can enhance the physical barrier effect of the coating. As nano-fillers, CDs can fill micropores and defects formed in the polymer coating during the curing process, prolonging the diffusion paths of water, oxygen, and corrosive ions (such as Cl^−^) (Figure 5a,b) and creating a “maze effect” [53,54,59,61,63]. For example, Pourhashem et al. [53] introduced silane-functionalized GQDs into epoxy, significantly improving the coating’s barrier performance. Dai et al. [61] and Zhu et al. [62] used CDs to intercalate α-ZrP, further strengthening the barrier effect of the lamellar material

Secondly, CDs enhance the density and adhesion of the coating through interfacial interactions. The abundant –OH, –COOH, –NH_2_, and other functional groups on the CDs surface can form covalent bonds, hydrogen bonds, or van der Waals forces with the polymer matrix (such as epoxy, polyurethane), thereby increasing the coating’s crosslinking density and adhesion to the metal substrate [52,54,57,65]. Kang’s group [52] pointed out that interfacial bonding between CDs and the polymer is the basis for achieving self-healing. Zhao et al. [54] found that the connection of NCDs with waterborne epoxy suppressed pore enlargement. Jin et al. [65] demonstrated that the polymer branches on the N-CD surface significantly increased the crosslinking density, thereby enhancing wear and corrosion resistance (Figure 5c).

Thirdly, CDs endow the coating with self-healing functions. Self-healing mechanisms mainly include two types: one is based on reversible interfacial bonding (such as hydrogen bonds, π-π interactions), allowing the coating to recombine after damage [52,57]; the other utilizes the responsiveness of CDs or their composites to corrosion products (such as Fe^3+^), forming complexes to inhibit corrosion propagation [2,58,66]. In the PCD-MX/WEP coating designed by Zhang et al. [58], the phenanthroline groups captured Fe^3+^ in situ to form [Fe(Phen)_3_]^2+^, which not only achieved self-healing but also inhibited the generation of corrosion products. Wang et al. [66] achieved thermally triggered repair through photothermal-responsive microcapsules.

Fourthly, CDs can exert corrosion inhibition or passivation effects. CDs can adsorb onto the metal surface, forming a protective adsorption film, or complex with metal ions to generate an insoluble passivation layer, hindering anodic dissolution [57,58,59,62]. For example, Zhao et al. [59] found that CDs anchored on LDH could coordinate with metal ions, promoting the formation of a passive layer. Zhu et al. [62] pointed out that P-CDs could adsorb onto the Q235 steel surface, forming a passive film and preventing further corrosion of the substrate.

In summary, the application of CDs in anticorrosive coatings has expanded from initial performance enhancement to the integration of multiple functions such as self-healing, monitoring, and lubrication, demonstrating broad application prospects. However, current research still faces challenges, such as the long-term stability of CDs in complex environments, large-scale low-cost preparation, and in-depth analysis of multi-functional synergistic mechanisms. The future design of high-performance anticorrosive coatings should focus on the multi-scale synergistic regulation of CDs with polymer matrices, two-dimensional materials, and other functional components to promote their application in practical engineering.

## 4. Progress on the Application and Mechanism of CDs in Photogenerated Cathodic Protection

Cathodic protection is an important electrochemical protection technique, based on the principle of suppressing corrosion by making the metal component the cathode in an electrochemical cell. Traditional methods are mainly divided into the sacrificial anode method and the impressed current method. Photogenerated cathodic protection is a green and sustainable cathodic protection technology that has emerged in recent years. Its core idea involves using a photoactive semiconductor material as a photoanode which, under illumination, generates photogenerated electron–hole pairs. The photogenerated electrons are transported through an external circuit to the metal surface requiring protection, causing cathodic polarization and shifting its potential into the thermodynamically stable region, thereby achieving protection. Meanwhile, the photogenerated holes are consumed at the photoanode surface by hole scavengers in the electrolyte. This technology can directly convert solar energy into the driving force for metal corrosion protection, holding great potential for energy saving and environmental protection. However, traditional photoanode materials like titanium dioxide (TiO_2_) are limited by their wide bandgap (approx. 3.2 eV), absorbing only UV light, and suffer from high photogenerated carrier recombination rates, restricting their photoelectric conversion efficiency and cathodic protection effect. CDs, owing to their unique advantages, including a tunable band structure, excellent up-conversion fluorescence properties (utilizing long-wavelength light), good electron conductivity/storage capacity, and abundant surface functional groups, have been introduced into the field of photogenerated cathodic protection. They aim to broaden the spectral response range of the photoanode, promote carrier separation, and enhance its cathodic protection performance for metals.

### 4.1. Construction and Application of CDs-Based Composite Photoanodes

Existing research mainly focuses on constructing heterostructure photoanodes by compositing CDs with wide-bandgap semiconductors like TiO_2_. In 2019, Du’s team [28] constructed a TiO_2_ nanotube array film on a Ti substrate via anodization, and then successively deposited silver nanoparticles and CQDs (with an average size of about 5 nm and a lattice spacing of 0.23 nm corresponding to the graphite (100) plane) using pulse current deposition and hydrothermal treatment, respectively, to prepare a CQDs/Ag/TiO_2_ composite film. The study showed that the synergistic sensitization effect of Ag NPs and CQDs significantly extended the light absorption range of the TiO_2_ composite film into the visible region. Under white light irradiation, the photocurrent density of this composite film in a 0.5 M Na_2_SO_4_ solution reached 110 mA cm^−2^, much higher than the 60 mA cm^−2^ of the pure TiO_2_ film. As a photoanode, the CQDs/Ag/TiO_2_ composite film exhibited significantly enhanced photogenerated cathodic protection, causing the potential of the coupled 403 stainless steel in a 0.5 M NaCl solution to drop by 400 mV relative to its corrosion potential. In 2020, Wang’s team [27] constructed a novel Al_2_O_3_-anchored CQDs/TiO_2_ nanorod array (Al/C/TNRs) photoanode via hydrothermal treatment and atomic layer deposition. This Al/C/TNRs photoanode achieved a photocurrent density of 2.28 mA cm^−2^ under simulated sunlight (AM 1.5G) and maintained it for up to 7 d with almost no decay. In a 3.5 wt.% NaCl solution, the potential of Q235 carbon steel was negatively shifted by 620 mV after coupling with Al/C/TNRs and could be maintained for over 7 h. This work fully demonstrated the potential of CDs-based composite photoanodes to provide efficient and stable photogenerated cathodic protection for carbon steel in marine environments.

It is worth noting that although research on the direct application of CDs in photogenerated cathodic protection is still relatively scarce, their use as components in constructing high-performance photoanodes has been well-validated, providing important support for further expansion in this direction. Studies have shown that CDs can be composited with semiconductors like TiO_2_, WO_3_, and ZnO to broaden the material’s response range to visible light, promote the separation and transport of photogenerated carriers, and improve interfacial reaction kinetics and photoanode stability [70,71,72,73]. For example, the CQDs/TiO_2_/WO_3_ composite photoanode constructed by Wang et al. exhibited enhanced light absorption and charge separation ability [70]. Zeng’s team demonstrated that CDs could act as excellent photosensitizers to enhance the photoelectric conversion performance of ZnO nanorod arrays [71]. Kang’s team further discovered that NCDs could significantly improve the photocurrent response of TiO_2_ photoanodes through an electron trap effect and impedance reduction [72]. Additionally, recent studies have shown that an S,NCDs layer can inhibit the chlorine evolution side-reaction and improve the corrosion resistance stability of photoanodes in seawater systems [73]. Although these studies are primarily oriented towards photocatalytic systems like water splitting, they collectively demonstrate that CDs possess significant advantages in enhancing light absorption, promoting charge separation/transfer, and improving service stability—precisely the key prerequisites for achieving efficient photogenerated cathodic protection.

### 4.2. Mechanisms of CDs in Enhancing Photoanode Performance

From existing research, the enhancing effects of CDs on photoanode performance are mainly manifested in the following aspects.

Firstly, CDs can significantly broaden the light absorption range of semiconductor materials, improving solar energy utilization efficiency [28]. Traditional semiconductors like TiO_2_ and ZnO typically respond strongly only to UV light. However, CDs, due to their abundant and tunable surface states and discrete energy levels, can generate multi-band absorption in the visible region. Notably, electronic transitions related to surface functional groups such as C=O and C-OH endow CDs with excellent visible light harvesting capability. When loaded onto the semiconductor surface, they can act as photosensitizers, converting more low-energy photons into usable excited electrons, thereby enhancing the overall photoresponse performance [28,71].

Secondly, CDs facilitate the separation and transport of photogenerated carriers, reducing electron–hole recombination. CDs typically form intimate interfacial coupling or heterojunction structures with semiconductors like TiO_2_, WO_3_, and ZnO, thereby establishing a favorable band alignment for directional charge migration [71,72]. On one hand, CDs can act as electron acceptors or intermediate transport mediators, rapidly extracting and transferring photogenerated electrons. On the other hand, some doped CDs can introduce electron trap states within the semiconductor, prolonging carrier lifetime and enhancing charge separation efficiency. For photogenerated cathodic protection, more efficient electron separation and output mean that more electrons can be transported to the protected metal surface, thereby achieving a more significant potential negative shift and superior protection effect [72].

Thirdly, CDs can regulate interfacial reaction kinetics and improve photoanode stability. Studies have shown that CDs modification not only reduces the bulk and interfacial charge transport resistance of the photoanode [72], but can also optimize the interfacial microenvironment through surface layer or composite layer structures, inhibiting unfavorable side reactions [73]. For example, in seawater systems, an N,SCDs splicing layer can isolate Cl^−^ from contact with TiO_2_ active sites, thereby inhibiting the chlorine evolution reaction and enhancing the corrosion resistance of the photoanode [73]. This implies that CDs are not merely “sensitizing components,” but could also serve as important functional layers for regulating selectivity and extending service life.

Finally, the synergistic effect between CDs and other functional components further amplifies their advantages. For instance, in the CQDs/Ag/ TiO_2_ system, Ag nanoparticles can work together with CQDs to enhance visible light response and electron transfer efficiency [28]. In the Al/C/TNRs system, the Al_2_O_3_ anchoring layer helps to enhance the stability and long-term operability of the composite structure [27].

In summary, the direct application of CDs in photogenerated cathodic protection is still in its infancy, with only a few publicly reported works, mainly focusing on TiO_2_-based composite photoanode systems. However, the immense potential of CDs in modifying photoanode materials has been fully demonstrated by a large body of related research. These studies have revealed the core mechanisms by which CDs broaden light absorption, promote carrier separation, and enhance stability, providing a solid theoretical foundation and feasible technological pathways for developing high-performance photogenerated cathodic protection systems. Nevertheless, key scientific issues such as the design of specialized CDs-based photoanodes for different metals (e.g., carbon steel, stainless steel, copper) in various corrosive media, the precise regulation of protection efficiency through the band structure of CDs, and the interfacial stability during long-term service currently lack systematic and in-depth investigation and urgently require exploration in future work. Furthermore, the future design of high-performance photoanodes for photogenerated cathodic protection should not be limited to simple CDs loading but should place greater emphasis on the multi-scale synergistic regulation of CDs with the semiconductor host, interfacial layers, and co-catalytic components.

## 5. Applications and Mechanisms of CDs in Other Corrosion Protection Sub-Fields

Beyond their demonstrated potential in mainstream areas such as corrosion inhibitors, anticorrosive coatings, and photogenerated cathodic protection, CDs, by virtue of its unique physicochemical properties, are also emerging in other relevant sub-fields of corrosion protection, opening up new application directions. On one hand, the low cost and high dispersibility of CDs lay a foundation for their application in cement-based materials. As nano-additives, CDs can regulate the cement hydration process, promote the formation of calcium silicate hydrate (C-S-H) gels and Friedel’s salt, thereby effectively enhancing the chloride binding capacity and intrinsically improving the corrosion durability of reinforced concrete structures. On the other hand, CDs possess excellent and tunable fluorescence properties. Their specific fluorescence quenching effects in response to particular ions (such as Fe^3+^, OH^−^) enable them to function as highly sensitive fluorescent probes for in situ, real-time monitoring of metal corrosion states or changes in the corrosive micro-environment. This chapter focuses on these two frontier directions, providing an in-depth review of the research progress, mechanisms, and development trends of CDs in the sub-fields of chloride binding promoters and corrosion sensing platforms.

### 5.1. CDs as Chloride Binding Promoters

Chloride-induced reinforcement corrosion is the predominant factor leading to the durability degradation of reinforced concrete (RC) structures. Enhancing the inherent chloride binding capacity of cement-based materials, converting free chlorides into bound states through physical adsorption (primarily on C-S-H gel surfaces) and chemical binding (forming Friedel’s salt, Fs), is a critical strategy to block chloride penetration for reinforcement and extending the service life of structures. However, traditional methods for enhancing chloride binding are either limited in efficiency or suffer from issues like high cost and poor dispersion of the introduced nanomaterials. As a novel zero-dimensional carbon-based nanomaterial, CDs, with their core advantages of low cost, excellent dispersibility, and abundant tunable surface functional groups, offer a new paradigm to address these challenges. In recent years, researchers have conducted systematic and in-depth work on the application of CDs in cement-based materials, revealing the efficacy and mechanisms of different types of CDs in enhancing chloride binding, and progressively establishing a clear research trajectory from “validating effectiveness” to “analyzing structure-activity relationships” and then to “expanding functional synergy.”

**Figure 6:**
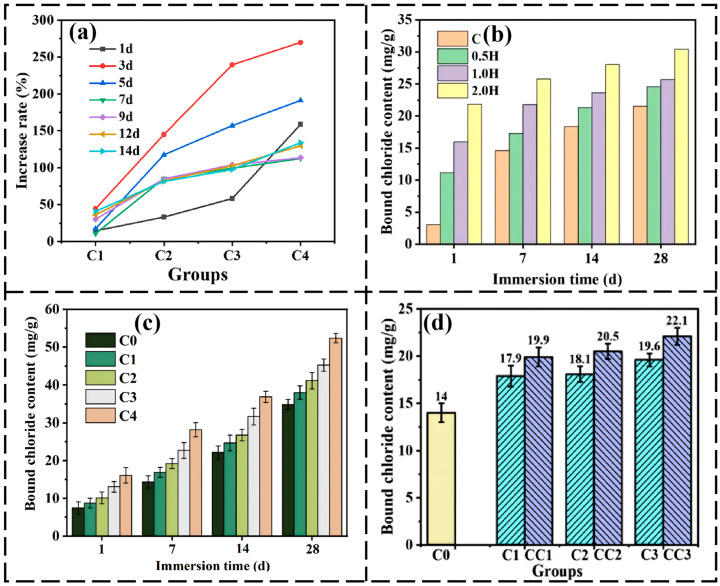
Chloride binding performance and hydration product evolution of cement-based materials modified with different types of CDs: (**a**) Enhancement rate of chloride binding capacity of cement modified with different dosages of NGQDs at ages of 1 to 14 d. All tests used blank cement paste without CDs as the control group. Reprinted with permission from Ref. [75]. Copyright 2023, Elsevier; (**b**) C_b_ values of various specimens at four exposure durations in the NaCl solution. Reprinted with permission from Ref. [77]. Copyright 2024, Elsevier; (**c**) Bound chloride content of various cement pastes. Reprinted with permission from Ref. [78]. Copyright 2026, Elsevier (**d**) Comparison of 14 d chloride binding capacity between CLDH/CDs composite systems with different dosages and the pure CLDH system. Reprinted with permission from Ref. [79]. Copyright 2024, Elsevier.

Pioneering work in this field was first conducted by He’s team in 2023 [74]. They designed and synthesized specific CDs via a hydrothermal method. The specific CDs are quasi-spherical and monodisperse with a size distribution of 1.0–4.6 nm (average ~2.63 nm), a lattice spacing of 0.21 nm corresponding to the graphene (100) plane, and a topographical height of 0.71–1.68 nm (confirmed by AFM), testifying their GQDs nature. XRD further revealed a peak at 27.2° assigned to the graphite (002) plane, indicating a highly crystalline sp^2^ carbon core. Additionally, the CDs possess abundant oxygen-containing functional groups. Incorporating 0.2 wt.% of these CDs into cement paste increased its chloride binding capacity by 109% compared to the blank group after exposure to NaCl solution, as confirmed by equilibrium tests. Mechanistic analysis revealed that the incorporation of CDs promoted cement hydration, generating more C-S-H gels and Friedel’s salt, thereby simultaneously enhancing both the physical adsorption and chemical binding of chloride ions. This work was the first to demonstrate the immense potential of CDs as efficient, highly dispersible nano-additives for improving the chloride corrosion resistance of RC structures. Building on these findings, the team further explored the enhancing effect of heteroatom doping. They synthesized Nitrogen-doped GQDs (NGQDs) via a one-step hydrothermal method [75]. The study found that 0.2 wt.% NGQDs dramatically boosted the chloride binding capacity of cement paste by 134% (Figure 6a), outperforming undoped CDs. The mechanism was primarily attributed to the significant enhancement of physical chloride adsorption by NGQDs, with a minor contribution to promoting chemically bound Friedel’s salt. This indicates that modulating the surface chemistry of CDs through nitrogen doping can effectively optimize their efficacy in enhancing chloride binding.

To promote practical applications, researchers have systematically investigated the relationships between the fundamental properties of CDs and their performance. He’s team synthesized extremely low-cost ($0.013/g) and highly dispersible citric acid/urea-derived CDs using a facile microwave method and systematically studied their effects on cement properties [76]. The results showed that 0.2 wt.% CDs increased chloride binding by 51%, but also significantly retarded early hydration. The mechanism revealed that the abundant surface groups on CDs complex with Ca^2+^, forming a protective barrier that delays hydration, while their nucleation effect later promotes the formation of C-S-H and Friedel’s salt, positively influencing chloride binding. Concurrently, He’s team focused on the critical parameter of CD size [77]. By controlling the calcination time of citric acid, they prepared CDs with different average sizes (1.73, 5.79, 12.37 nm). Their study found a negative correlation between CD size and chloride binding capacity: smaller CDs (1.73 nm) yielded the most significant enhancement (41%). The mechanism is that smaller CDs provide more nucleation sites, more effectively promoting cement hydration and the generation of C-S-H and monosulfate, thus more markedly enhancing chloride binding. These studies provide crucial guidance for tailoring CD properties to optimize their performance (Figure 6b).

Researchers have also continuously expanded the sources of CD materials [30,78,79,80,81]. For example, Chuang He’s team was the first to introduce polymer dots (PDs), prepared at room temperature via Schiff base reaction, into cement-based materials [81]. Incorporating 0.2 wt.% PDs enhanced chloride binding by 106% while reducing the water absorption of the hardened paste. The mechanism was attributed to the synergistic enhancement of physical adsorption and chemical binding, along with improved resistance to chloride migration within the matrix. Pursuing true green and sustainable approaches, He’s team used renewable and environmentally friendly biomass waste (e.g., litchi leaves) as a precursor to prepare leaf-derived CDs [78]. A 0.2 wt.% dosage increased chloride binding by 59.34% after 28 d of immersion in 3 M NaCl solution (Figure 6c). Meanwhile, Zhou et al. explored a “waste control for waste” strategy by synthesizing CDs from the pharmaceutical waste norfloxacin [80]. These CDs not only improved the mechanical properties and impermeability of cement but, importantly, facilitated the phase transformation of cement minerals towards Friedel’s salt, boosting the chloride binding capacity by 62.1%. These efforts not only expand green synthesis pathways for CDs but also achieve resource utilization of waste materials.

CDs are not only effective on their own, but can also act as dispersants to enhance the application of other functional materials. He’s team found that calcined layered double hydroxide (CLDH), despite its excellent chloride adsorption potential, tends to agglomerate in cement matrices. They innovatively composited CDs with CLDH to prepare highly dispersed CLDH/CDs composites [79]. The introduction of CDs increased the specific surface area of CLDH by 166.4%, and a mere 0.8 wt.% of the composite enhanced the chloride binding capacity of cement by 52.41% (Figure 6d). The enhancement mechanism stems from the composite providing nucleation sites to accelerate hydration while leveraging CLDH’s inherent structure memory effect and anion exchange adsorption capacity, achieving a “1 + 1 > 2” synergistic effect.

In summary, CDs, as a novel class of nano-additives, demonstrate significant advantages and broad prospects in enhancing the chloride binding capacity of cement-based materials. The research trajectory is clear and in-depth, progressing from initial feasibility validation, through performance optimization via doping and size control, to the exploration of green precursors and synergistic applications. The core mechanisms can be attributed to the nano-nucleation effect of CDs (promoting the formation of C-S-H and Friedel’s salt, enhancing physical adsorption and chemical binding) and their abundant surface functional groups (complexing with Ca^2+^ to regulate hydration or acting as reactive sites). These studies pave new technological pathways for developing high-performance and long-lasting reinforced concrete structures.

### 5.2. CDs as Corrosion Sensing Platforms

The complete elimination of corrosion is unattainable; therefore, achieving rapid and visual detection at the early stages of corrosion is of great significance for timely intervention, preventing structural material failure, and ensuring engineering safety [82]. Traditional corrosion monitoring techniques, such as weight loss methods and electrochemical measurements, often suffer from limitations including slow response, difficulty in achieving in situ online monitoring, and inability to provide visual information. CDs, with their excellent and tunable fluorescence properties, good photostability, low toxicity, and easily functionalizable surface groups, offer new solutions to address these challenges. Notably, the specific fluorescence response (typically quenching or recovery) of CDs to particular ions (such as Fe^3+^, Fe^2+^, OH^−^, H^+^) enables them to function as highly sensitive fluorescent probes. They can convert characteristic ions generated during corrosion or localized microenvironmental changes into intuitive fluorescence signals, thereby achieving early, in situ, and visual corrosion monitoring. In recent years, researchers have conducted a series of studies around this application direction of CDs, progressively building a research framework from “integrated inhibition-monitoring dual functionality” to “innovative detection mechanisms.”

Integrating corrosion protection and corrosion monitoring functions within the same material system is a crucial strategy for achieving intelligent corrosion control. Jia’s team conducted pioneering exploration in this direction [83]. They synthesized fluorescent nitrogen-doped CDs via a simple solvothermal method using citric acid as the carbon source and ammonia as the dopant. The obtained CDs were well dispersed, uniform spherical particles with a size distribution of 2–8 nm (average 5.3 nm, TEM). FTIR and XPS revealed surface groups including –OH, –COOH, C–N, and C=O, with nitrogen content of 5.56 at%. XRD showed a broad peak at 20° (amorphous graphite), and Raman exhibited D and G bands at 1350 and 1560 cm^−1^, confirming a disordered graphitic structure with sp^2^ domains. The study found that the active groups on the CD surface promoted the formation of an adsorption film on iron plates while maintaining effective fluorescence emission, thereby endowing the CDs with dual functions of corrosion inhibition and corrosion monitoring. At a concentration of 180 mg/L, the CDs achieved an inhibition efficiency of 88% for iron. More importantly, as corrosion intensified, the concentration of Fe^3+^ released from the iron matrix increased. The complexation between CDs in the adsorption film and Fe^3+^ led to sensitive and visual fluorescence quenching. This “inhibition–monitoring” synergistic strategy provided a new paradigm for tracking and intervening in early-stage corrosion. The team further validated the response performance of CDs to Fe^3+^, finding a detection limit as low as 0.9 μM, a linear response range of 10–300 μM, and fluorescence quenching visible to the naked eye [84]. Based on this, they loaded CDs into cellulose and polyvinyl alcohol matrices to fabricate patterned paper and hydrogels, successfully using them for visual detection of iron corrosion, offering a simple and intuitive strategy for safety monitoring of structural metal materials.

Building on the dual-function concept, researchers further enhanced the comprehensive performance of CDs through chemical modification and material design. Ma’s team synthesized a sulfosalicylic acid-modified CD complex (CDs-SSA) [85]. Electrochemical tests and surface characterization showed that, compared to unmodified CDs, SSA or the CDs–SSA complex significantly improved corrosion inhibition performance for carbon steel in HCl solution, exhibiting anodic passivation behavior and greatly increased impedance modulus, achieving a maximum inhibition efficiency of 99.1% at 200 mg/L after 24 h of immersion. The mechanism involves CDs–SSA forming a hydrophobic physicochemical adsorption film that prevents corrosive media attack. DFT and MD simulations further revealed that the CDs–SSA complex, with abundant active sites, adsorbs on the metal surface in a parallel manner, achieving maximum coverage and the highest binding energy. Concurrently, CDs–SSA exhibited sensitive fluorescence quenching responses to both Fe^2+^ and Fe^3+^ ions, achieving the synergistic integration of efficient inhibition and sensitive monitoring. Cui’s team approached from the perspective of carbon source selection, preparing nitrogen-doped CDs via a hydrothermal route using L-phenylalanine and L-tryptophan (1:1 molar ratio) as precursors [86]. These NCDs exhibited excellent corrosion inhibition for carbon steel in 1 M HCl solution (96.04% efficiency at 200 ppm), with the Langmuir adsorption isotherm revealing an adsorption mechanism involving both physisorption and chemisorption. Crucially, these NCDs also displayed pronounced fluorescence quenching towards Fe^3+^ ions, enabling early-stage detection of corrosion onset. These works demonstrate effective pathways for optimizing the dual functionality of CDs through molecular design and precursor screening.

Beyond monitoring corrosion product ions, researchers have explored the response mechanisms of CDs to changes in the corrosive micro-environment. Baorong Hou’s team developed a green “off-on” fluorescence sensor based on phytic acid-derived carbon dots (PACDs) for the detection of OH^−^ and H^+^ during metal corrosion [87]. The study found that the fluorescence of PACDs gradually turned “off” (quenched) as the OH^−^ concentration increased from 20 to 340 μmol/L; conversely, under ultrasonic treatment, the fluorescence gradually turned “on” (recovered) as the H^+^ concentration increased from 60 to 230 μmol/L. The detection limits for OH^−^ and H^+^ were estimated at 7.33 μmol/L and 20.34 μmol/L, respectively. Mechanistic studies attributed this “off-on” behavior to the aggregation and disaggregation of nanoparticles induced by the deprotonation and protonation of -H_2_PO_4_ functional groups on the PACD surface. After loading PACDs onto a film and using it for Q235 steel packaging, the fluorescence “turn-off” phenomenon could be visually observed upon the onset of steel corrosion, demonstrating the potential application of PACD films for early-stage corrosion monitoring. This work expanded the monitoring targets of CDs from corrosion products (Fe^3+^) to the corrosive micro-environment (pH changes), enriching the mechanistic dimension of CD-based corrosion sensing.

The fluorescence properties of CDs can not only be used for corrosion monitoring but also, conversely, to study the adsorption behavior of CDs themselves, deepening the understanding of inhibition mechanisms. The authors of [88] synthesized biomass-derived N, Br co-doped carbon dots (HCDs), which achieved over 97% inhibition efficiency for carbon steel in a hydrochloric acid environment at 150 mg/L, with excellent durability (120 h). More innovatively, they capitalized on the intrinsic fluorescence of HCDs, employing them as fluorescent probes for corrosion monitoring and adsorption behavior tracking. The results revealed that HCDs possess strong affinity, ultra-sensitivity (LOD: 0.274 μM), and a broad detection range towards Fe^3+^. Crucially, by monitoring changes in fluorescence signals, they successfully tracked the adsorption behavior of HCDs on the metal surface: initially transient adsorption followed by equilibrium adsorption. This not only unveiled the strong interaction between CDs and the metal surface, but also provided a fresh perspective for exploring the mechanisms of CDs as corrosion inhibitors.

In summary, CDs, as a novel class of fluorescent nanomaterials, demonstrate unique advantages and broad prospects in the field of corrosion detection. The research trajectory has progressively deepened: from the initial validation of the “inhibition–monitoring” dual-function concept, through performance optimization via chemical modification and material design, to the innovative expansion of detection mechanisms (from monitoring Fe^3+^ to monitoring pH changes), and the reverse application of fluorescence properties to study adsorption behavior. The core mechanism lies in the abundant functional groups on the CD surface, enabling specific interactions with characteristic ions (e.g., Fe^3+^, Fe^2+^) generated during corrosion or environmental changes (pH), leading to alterations in fluorescence signals. Currently, research in this field is evolving from mere “detection” towards “integrated detection–protection” and “detection–mechanism study coupling,” providing powerful tools for achieving intelligent corrosion monitoring and control of metallic materials.

## 6. Conclusions and Outlook

### 6.1. Conclusions

CDs, as an emerging class of zero-dimensional carbon-based nanomaterials, exhibit broad application prospects in the field of corrosion protection by virtue of their small size, excellent dispersibility, abundant and tunable surface functional groups, low cost, environmental friendliness, and unique fluorescence properties. This study systematically reviewed the application progress and mechanisms of CDs in the fields of corrosion inhibitors, anticorrosive coatings, photogenerated cathodic protection, as well as chloride binding promoters and corrosion sensing platforms. The main conclusions are as follows:

In the field of corrosion inhibitors, CDs have demonstrated excellent protection performance for various metals such as carbon steel and copper in acidic, neutral/saline, and alkaline media. The inhibition mechanism primarily originates from the synergistic effect of physical and chemical adsorption of CDs on the metal surface, forming a dense protective adsorption film that effectively blocks the attack of corrosive media. Heteroatom doping (especially N and S doping) and the selection of biomass precursors are key strategies for optimizing the inhibition performance of CDs and achieving green and sustainable development. Nitrogen doping enhances the electron-donating ability of CDs, thereby strengthening chemisorption; multi-element co-doping provides multiple adsorption sites, achieving multi-anchoring effects. In recent years, the introduction of machine learning methods has provided a new paradigm for optimizing synthesis conditions and predicting the performance of CD-based inhibitors, which is expected to significantly shorten the research and development cycle and reduce costs.

In the field of anticorrosive coatings, the introduction of CDs not only significantly enhances the physical barrier effect and interfacial adhesion of the coating, but also endows the coating with multiple intelligent functions such as self-healing, corrosion monitoring, lubrication, and photothermal response. As nano-fillers, CDs can fill micropores and defects in the coating, prolonging the diffusion path of corrosive media. The abundant functional groups on their surface form interactions such as covalent and hydrogen bonds with the polymer matrix, increasing the crosslinking density and adhesion of the coating. Self-healing functions are mainly achieved through reversible interfacial bonding or the responsive complexation of CDs with corrosion products (e.g., Fe^3+^). The synergistic modification of CDs with two-dimensional materials (such as graphene, MXene, LDH, α-ZrP) further amplifies their protective effect in coatings, achieving a “1 + 1 > 2” synergistic enhancement.

In the field of photogenerated cathodic protection, although direct application research on CDs is still in its infancy, the immense potential of CDs in modifying photoanode materials has been fully demonstrated by numerous related studies. By compositing with wide-bandgap semiconductors such as TiO_2_, WO_3_, and ZnO, CDs can significantly broaden the light absorption range of the photoanode to the visible region, promote the separation and transport of photogenerated carriers, reduce interfacial charge transfer resistance, and regulate interfacial reaction kinetics, inhibiting unfavorable side reactions (such as the chlorine evolution reaction in seawater systems). The synergistic effect of CDs with functional components like Ag nanoparticles and Al_2_O_3_ anchoring layers further enhances the stability and long-term serviceability of the photoanode. These mechanisms collectively lay a theoretical foundation for achieving efficient and stable photogenerated cathodic protection.

In other corrosion protection sub-fields, CDs demonstrate unique value in functional expansion. As chloride binding promoters, CDs promote the formation of cement hydration products (C-S-H gels and Friedel’s salt) through their nano-nucleation effect, simultaneously enhancing the physical adsorption and chemical binding of chloride ions, thereby intrinsically improving the chloride corrosion resistance of reinforced concrete structures. The size, doping type, and precursor source (especially biomass waste) of CDs significantly influence their binding efficiency, and CDs can also act as dispersants to assist other functional materials (e.g., CLDH) in achieving synergistic effects. As corrosion sensing platforms, CDs utilize their specific fluorescence response to characteristic species such as Fe^3+^, Fe^2+^, OH^−^, and H^+^ to achieve early, in situ, and visual corrosion detection. The development of “inhibition–monitoring” dual-functional CDs, and the mechanistic expansion from monitoring corrosion products to monitoring the corrosive micro-environment (pH changes), provide powerful tools for intelligent corrosion control. The fluorescence properties of CDs can also be reversely used to track their own adsorption behavior on metal surfaces, deepening the understanding of inhibition mechanisms.

### 6.2. Future Research Outlook

Although significant progress has been made through research on CDs in the field of corrosion protection, this area is still in a stage of rapid development, and numerous key scientific issues and application challenges urgently need to be addressed. Future research can be explored from the following aspects:

(1) Controllable Synthesis and Scalable Preparation of CDs

Currently, various methods exist for synthesizing CDs, but most remain at the laboratory scale, suffering from low yield and poor batch-to-batch reproducibility, which hinders industrial application. Beyond scalability, cost-effectiveness is a critical issue for practical applications beyond the laboratory level. Although some studies have reported a cost reduction of carbon dots for corrosion protection to approximately $10/kg [33,76], such results are achieved only at laboratory scale and consider solely raw material costs, excluding labor and other associated expenses. Future efforts should focus on developing green, low-cost, and scalable synthesis processes to achieve precise control over the size, morphology, doping type, and surface functional groups of CDs. Concurrently, establishing a structure–activity relationship database correlating CD structure with performance is necessary to provide theoretical guidance for the targeted design of high-performance CDs.

(2) Long-Term Service Performance and Stability in Complex Environments

Existing research primarily focuses on short-term performance validation, with a lack of systematic understanding regarding the long-term stability, aging resistance, and degradation mechanisms of CD performance in complex corrosive environments (e.g., high temperature, high pressure, strong acids/bases, coexistence of multiple ions, microbial activity). Future efforts should involve long-term performance evaluation under simulated real-world conditions to reveal the structural evolution patterns and failure mechanisms of CD-based materials under environmental stressors, providing a reliable basis for their practical application.

(3) Structure-Activity Relationship Studies of CDs

A deep understanding of the structure-activity relationship of CDs is the theoretical foundation for achieving precise control over their performance. The core structural characteristics of CDs, including the sp^2^/sp^3^ hybridization ratio of the carbon core, graphitization degree, defect density and size, as well as the type, quantity, distribution of surface functional groups, and the configuration of doping atoms (N, S, P, etc.), collectively determine their behavior in corrosion protection. For instance, in the field of inhibition, the crystallinity of the carbon core synergistically influences adsorption strength and film compactness with surface functional groups. In photoanode applications, the band structure, up-conversion fluorescence properties, and electron mobility of CDs directly determine their light absorption range and carrier separation efficiency. Future research should combine high-resolution characterization techniques with theoretical calculations to systematically establish the intrinsic correlation between the structural features of CDs and their protective performance, providing a scientific basis for the targeted design of CD materials for different application scenarios.

(4) In-Depth Elucidation of Multi-Factor Coupling Mechanisms

The role of CDs in corrosion protection often results from the synergy of multiple mechanisms, including physical barriers, chemical adsorption, interfacial regulation, and electrochemical effects. The current understanding of the coupling between these mechanisms remains ambiguous, making precise control difficult. Moreover, it remains unclear whether the inhibitory effect of CDs originates from the carbon dot carrier itself or from the modifying compounds (e.g., surface functional groups, heteroatom dopants, or grafted molecules) that may possess inherent inhibition properties. This distinction is particularly critical because carbon materials, when in contact with metals and alloys, typically act as cathodes and could potentially accelerate corrosion processes, a risk that must be carefully evaluated. Future research should combine advanced in situ characterization techniques (e.g., in situ spectroscopy, electrochemical atomic force microscopy) with multi-scale theoretical simulations (density functional theory, molecular dynamics, finite element analysis) to deeply unravel the nature of interactions between CDs and metal surfaces, corrosive media, and polymer matrices at the atomic/molecular level, thereby elucidating the protection mechanisms under multi-factor coupling. Meanwhile, systematic comparative studies are urgently needed to decouple the intrinsic role of the CD core from that of surface functional groups or modifying compounds, clarifying whether the observed protection arises from the CDs themselves or from the release/adsorption of active modifying species.

(5) Machine Learning-Assisted Design of High-Efficiency CDs

Data-driven machine learning methods offer powerful tools for the rapid screening and performance prediction of CD-based materials. Future efforts should focus on further enriching and standardizing experimental data in the field of CDs for corrosion protection, constructing high-quality, standardized dedicated databases. Building upon this, developing interpretable machine learning models will allow for deep mining of the implicit correlations between CD composition, structure, synthesis parameters, and protective performance, enabling a paradigm shift from “trial-and-error screening” to “design-on-demand” and accelerating the R&D process for novel high-efficiency CD materials.

(6) Construction of Multifunctional Integrated Intelligent Protection Systems

Single-function CD materials are increasingly insufficient to meet the diverse demands of corrosion protection in complex service environments. Future efforts should prioritize the development of multifunctional intelligent coatings or inhibitor systems integrating “protection–monitoring–repair–warning” capabilities. For example, combining CDs with corrosion inhibition functions and fluorescence sensing properties enables in situ early warning of corrosion initiation and autonomous repair. Compositing CDs with photothermal responsive materials allow for remotely triggered self-healing. Incorporating CDs into self-healing microcapsule systems facilitates multiple autonomous repairs. Through multi-scale, multi-component synergistic design, the next generation of intelligent corrosion control systems capable of active sensing, intelligent response, and sustained protection can be constructed.

In summary, CDs have been widely explored in diverse fields such as biomedicine, optical devices, chemical sensing, catalysis, lubrication, and agriculture. Nevertheless, their use in these areas remains largely confined to the laboratory stage, with limited translation into practical engineering applications. Consequently, CDs have yet to demonstrate proven utility beyond the laboratory scale. Corrosion protection emerges as a particularly promising breakthrough direction for advancing CDs toward real-world deployment. If the production cost of CDs can be further reduced and their corrosion protection mechanisms are better elucidated, their practical application in corrosion protection will become increasingly feasible. Given their unique advantages, CDs hold great potential for developing next-generation, high-performance, and sustainable corrosion protection systems.

## Data Availability

No new data were created or analyzed in this study. Data sharing is not applicable to this article.
